# 001 Egyptian consensus-based recommendations for the diagnosis and targeted management of Kawasaki disease. An initiative by the Egyptian College of Pediatric Rheumatology

**DOI:** 10.1093/rheumatology/keac495.001

**Published:** 2022-10-07

**Authors:** Y El Miedany, H Lotfy, S Salah, M. H Abu-Zaid, S. S Mohamed, S Esam Maher, M El Gaafary, H Abdulhady, W. A Hassan, M Mortada, Y Amer, N. S Osman, B. M Medhat, Y Farag, M Eissa, A Radwan, S. I Nasef, N. E Elkaraly, Amira T El-Shanawany, D El Mikkawy, D. M Mosa, G El Deriny, N. A Fouad, S. A Tabra

**Affiliations:** 1 Canterbury Christ Church University, England; 2 Rheumatology and Rehabilitation, Ain Shams University, Egypt; 3 Pediatric Rheumatology, Cairo, University, Egypt; 4 Rheumatology and Rehabilitation, Tanta University, Egypt; 5 Rheumatology and Rehabilitation, Cairo University, Egypt; 6 Pediatric Rheumatology, Minia University, Egypt; 7 Community medicine and Public Health, Ain Shams University, Egypt; 8 Rheumatology and Rehabilitation, Benha University, Egypt; 9 Rheumatology and Rehabilitation, Zagazig University, Egypt; 10 Pediatric Rheumatology, Assuit University, Egypt; 11 Rheumatology and Rehabilitation, Sohag University, Egypt; 12 Rheumatology and Rehabilitation, Suez Canal University, Egypt; 13 Rheumatology and Rehabilitation, Menoufia University, Egypt; 14 Rheumatology and Rehabilitation, Mansoura University, Egypt; 15 Pediatric Rheumatology, Alexandria University, Egypt; 16 Rheumatology and Rehabilitation, Fayoum University, Egypt

## Abstract

**Background:**

Kawasaki disease (kDa) is a self-limiting acute vasculitis that affects small and medium-sized vessels, and is the most common cause of acquired heart disease in children. It is also an important reason for long-term cardiac disease into adulthood. Rapid diagnosis and management of kDa is challenging due to the heterogeneity of the disease, yet is vital for improving outcome. To date, there are no Egyptian nationally agreed, evidence-based guidelines concerning the diagnosis and management of kDa in children. Consequently, treatment regimens vary broadly.

**Objectives:**

To develop a consensus, evidence-based recommendations for the diagnosis, evaluation and management of children living with kDa.

**Methods:**

This study was carried out to achieve an Egyptian expert consensus on a management strategy for kDa using Delphi technique. A multistep process strategy was adopted, which started by developing 16 key clinical questions by scientific committee according to the Patient/Population, Intervention, Comparison, Outcomes and Time (PICOT) approach. The core leadership team identified clinicians and researchers with expertise in pediatric rheumatology all over Egypt. An evidence-based, systematic, literature review was conducted to compile evidence for the kDa management. Delphi process was implemented (3-rounds) to reach a consensus.

**Results:**

Twenty-five expert panel participated in the 3 rounds with response rate 100%. A total of 21 recommendations, categorized into 9 domains (Definition, disease activity, predicting the development of coronary disease, assessment and monitoring (lab, imaging), treatment (acute and after acute attack), management of resistant cases, management of complications (cardiac complications, MAS and arthritis), vaccination and long term follow up. The Agreement with the recommendations (rank 7–9) ranged from 83.6–95.7%. The Consensus was reached (i.e. ≥75% of respondents strongly agreed or agreed) on all the clinical standards. Algorithm for management has also been developed.

**Conclusion:**

This was an expert, consensus recommendations for the diagnosis and treatment of kDa, based on best available evidence and expert opinion. The recommendations provided a management approach based on easy-to-use algorithm and with the support of complementary tests.

**The implication to policy, practice, research and advocacy:**

to provide updated recommendations for better management of kDa.

